# Supplementary material for: Ultrastructure of macromolecular assemblies contributing to bacterial spore resistance revealed by in situ cryo-electron tomography
Source: Nat Commun. 2024 Feb 14;15:1376. doi: 10.1038/s41467-024-45770-6 (PMC10867305; doi:10.1038/s41467-024-45770-6)
Supplement: Supplementary file 1 — Supplementary Information [file 41467_2024_45770_MOESM1_ESM.pdf]

SUPPLEMENTARY INFORMATION

Ultrastructure of macromolecular assemblies contributing to bacterial spore resistance revealed by in situ cryo-electron tomography

Elda Bauda, Benoit Gallet, Jana Moravcova, Gregory Effantin, Helena Chan, Jiri Novacek, Pierre-Henri Jouneau, Christopher D.A. Rodrigues, Guy Schoehn, Christine Moriscot and Cecile Morlot

Other online supporting materials for this manuscript include the following:
Supplementary Movies 1 to 4

SUPPLEMENTARY METHODS

Cryo-FIBM of sporulating *B. subtilis* cells for cryo-electron tomography

Since cryo-FIBM/ET still remains an emerging technique, we have not found any publication that would thoroughly describe preparation of *B. subtilis* samples for cryo-FIBM. We thus tested several parameters to obtain reproducible, thin and robust lamellae of sporulating *B. subtilis* cells. The quality of the prepared lamellae was mostly influenced by the cell concentration, blotting conditions to obtain a uniform monolayer of cells, the vitrification procedure and the milling protocol. Low cell concentration (below OD_{600nm} ~ 5) resulted in isolated cells sparsely distributed on the EM grid, while high cell concentration (OD_{600nm} ~ 20) allowed an even distribution of cells on the grid surface. To obtain a 5 to 10 µm-thick uniform layer of cells (Supplementary Fig. 4a), we blotted the back side of the grid with filter paper, and the cell side with a non-absorbent, granular, home-made plastic pad. Importantly, blotting with this device, which exhibited irregularities at its surface, preserved the integrity of the grid better than using smooth plastic polymers like Teflon® pads. The ethane phase had also a notable impact on the stability of the lamellae. We observed that plunge freezing performed at the limit of solidification (milky appearance of ethane), rather than in full-liquid phase (transparent ethane), favored proper sample vitrification. To relieve the tension on the lamellae during and after cryo-FIBM, we milled two trenches on both sides of the lamella (Supplementary Fig. 4b-c) [1]. Finally, the last step of polishing was crucial to remove redeposited material from the milling of neighboring lamellae [2]. The best lamellae were obtained using the protocol described in the *Online Methods* section. The lamellae thickness was in the range of 150 to 200 nm, as estimated from reconstructed tomograms.

SUPPLEMENTARY FIGURES

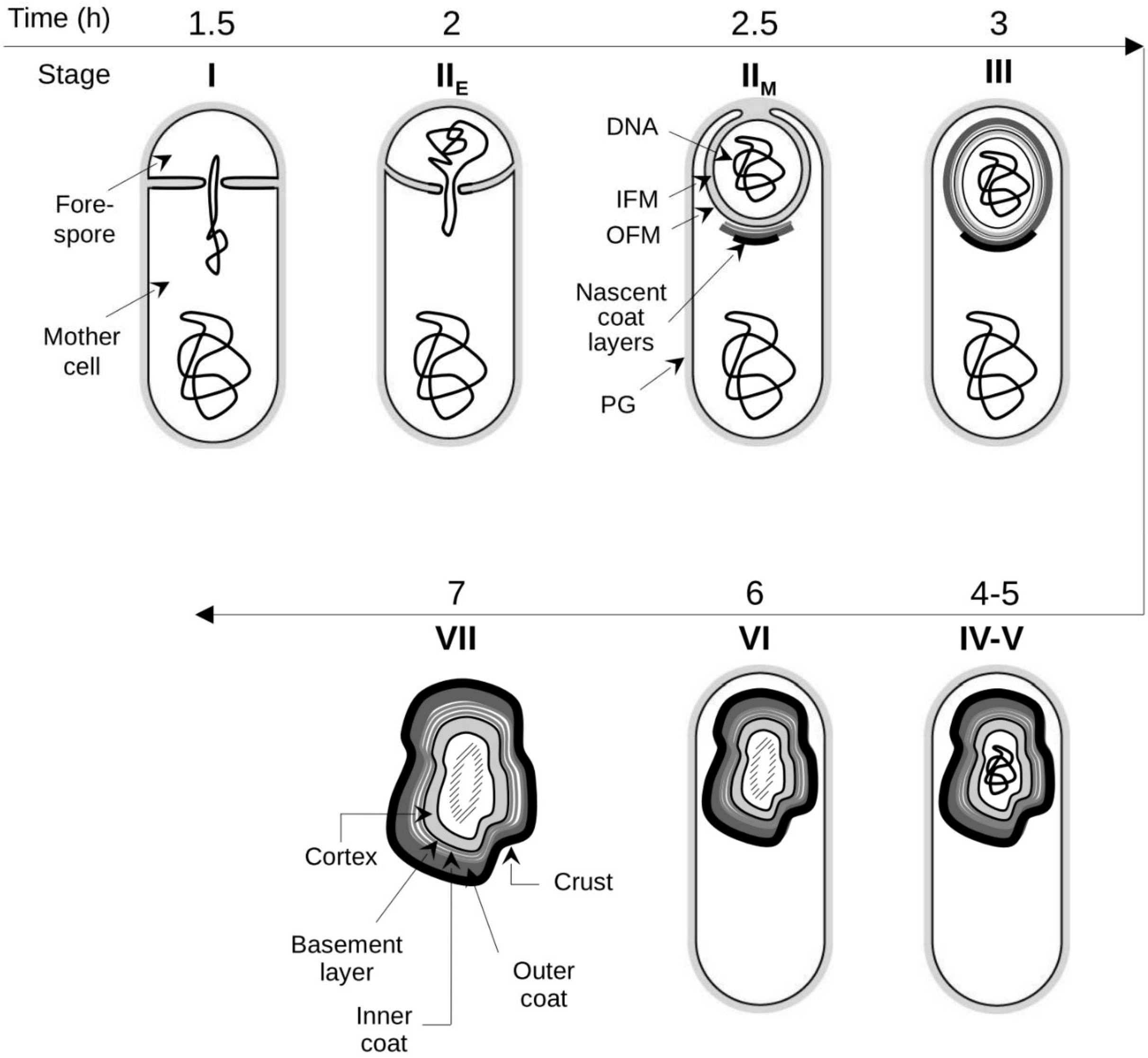

Supplementary Figure 1. Schematic illustration of main ultrastructural changes occurring during the different stages of sporulation. IFM, inner forespore membrane; OFM, outer forespore membrane; PG, peptidoglycan; II$_E$, early-engulfment; II$_M$, mid-engulfment.

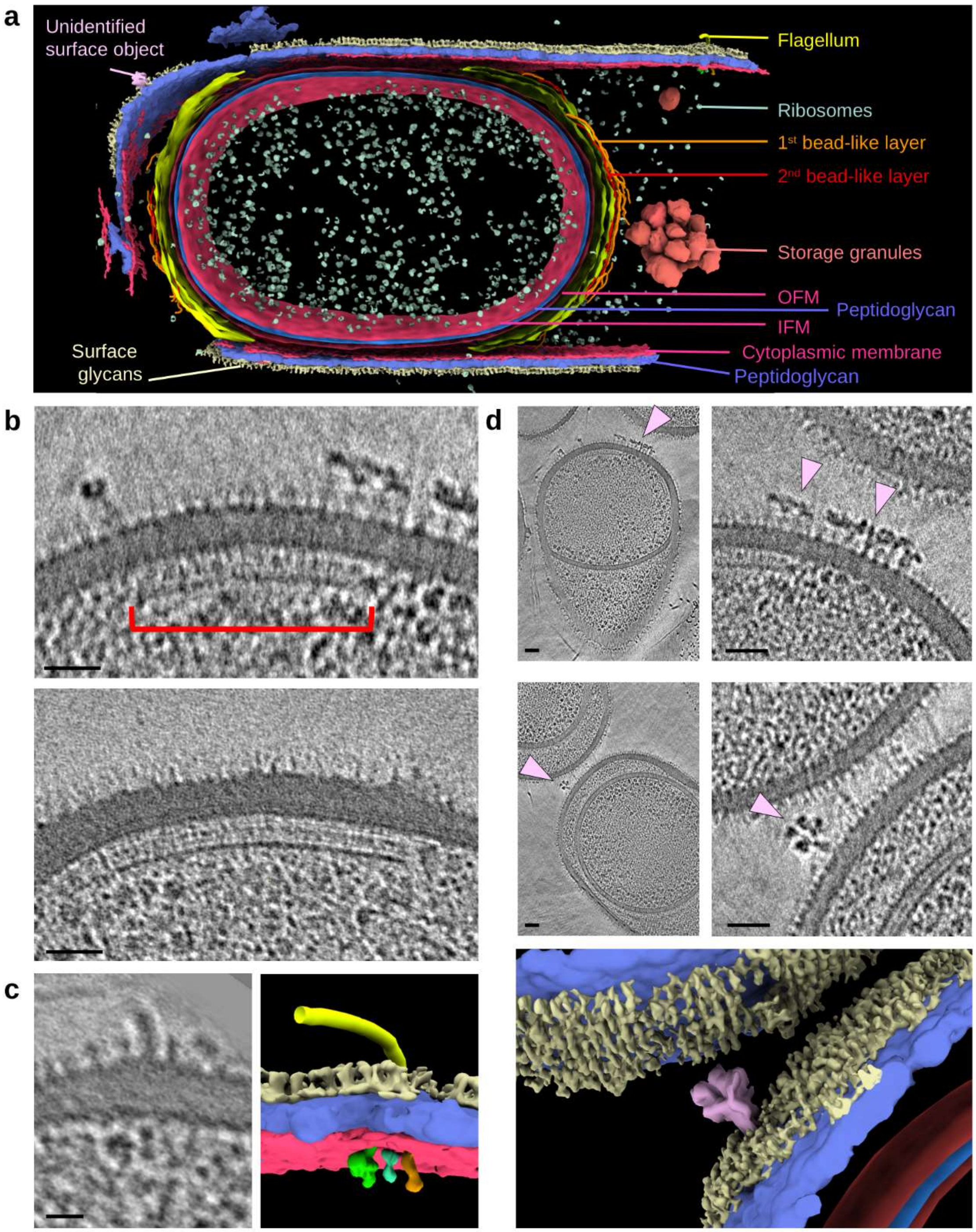

Supplementary Figure 2. Envelope-associated objects revealed by cryo-FIBM/ET. **a.** Segmentation of various forespore and mother cell ultrastructures performed from the tomogram slice of a stage-III *ΔspoIVB B. subtilis* sporangium, shown in Fig. 1a. **b-d.** Magnified views of tomogram slices showing chemotaxis arrays (**b**, red bracket, scale bars = 50 nm), a flagellum (**c**, scale bar = 20 nm) and unidentified surface elements (**d**, pink arrowheads, scale bars = 50 nm). Segmentation is shown for the surface components with the same color code than in panel a. The images are representative of 2 independent experiments, with 9 (**b**), 2 (**c**) and 18 (**d**) cells displaying similar features.

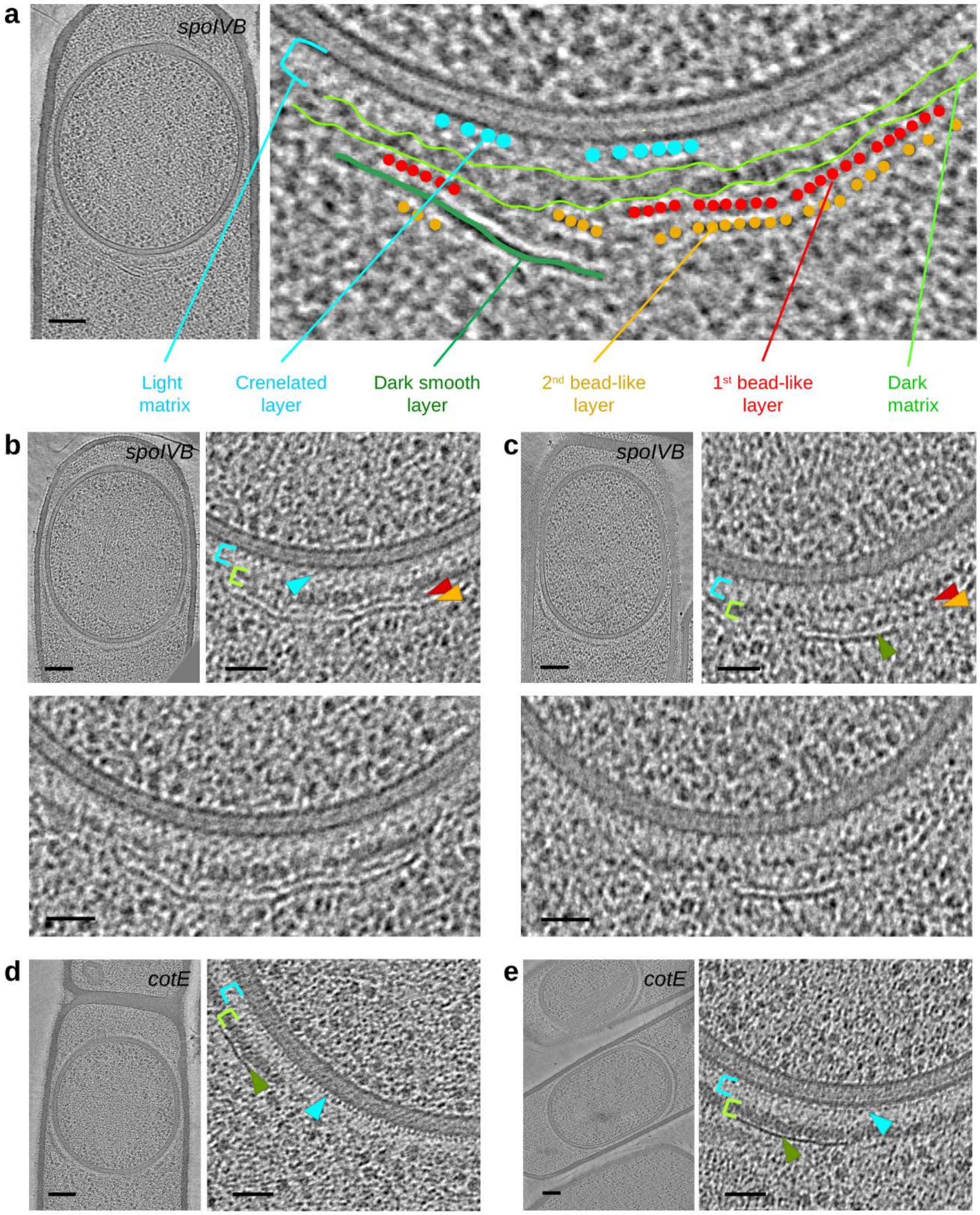

Supplementary Figure 3. Slices through cryo-electron tomograms of *ΔspoIVB* (**a-c**) and *ΔcotE* (**d-e**) sporangia, shown in full view (scale bars = 100 nm) or as magnified views of specific regions (scale bars = 50 nm). Ultrastructures corresponding to the crenelated layer (cyan beads or cyan arrowhead), the light matrix (cyan bracket), the dark matrix (lime lines or lime bracket), the two bead-like layers (red and orange beads or red and orange arrowheads) and the dark smooth layer (green line or green arrowhead) are indicated. For each strain, the images are representative of 2 independent experiments, with 7 (**a**), 15 (**b**), 7 (**c**) and 6 (**d-e**) cells displaying similar features.

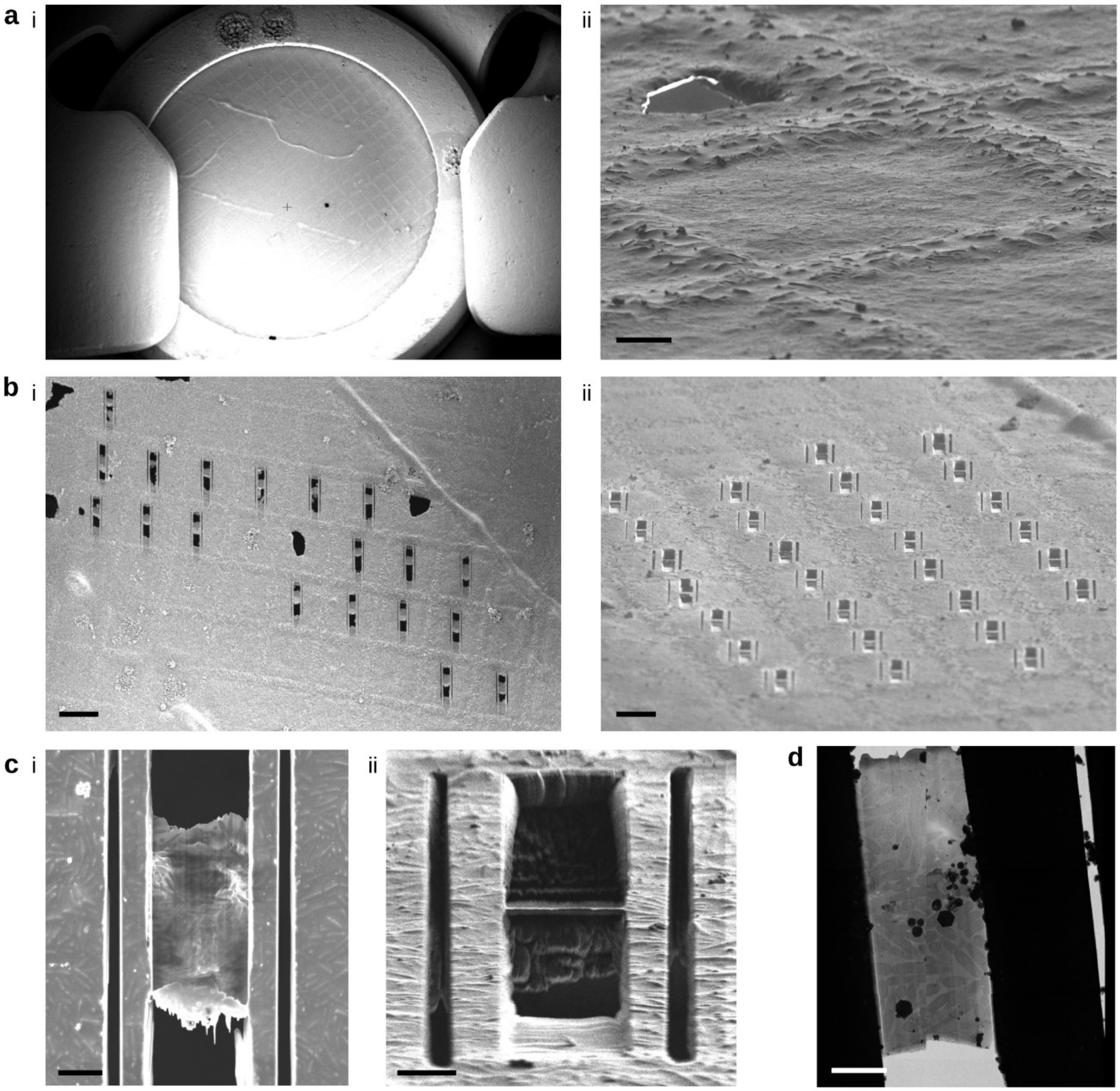

Supplementary Figure 4. a. Full (**i**) and close (**ii**, scale bar 5 µm) views of a grid covered with a homogenous layer of *B. subtilis* cells, observed by SEM and FIB, respectively. **b.** Lamellae of vitrified cells milled by FIB, observed from the SEM (**i**) and FIB (**ii**) perspective. Scale bars = 50 µm. **c.** Zoom on a FIBM lamella observed from the SEM (**i**) and FIB (**ii**) perspective. Scale bars = 5 µm. **d.** TEM view of a FIBM lamella containing vitrified *B. subtilis* cells. Scale bar = 5 µm. The images are representative of about 60 independent experiments.

Supplementary Table 1. Strains used in this study.

Construct	Genotype	Source
***B. subtilis* strains**		
BDR2413	168	[3]
BCR1661	168, *spoIVB::spec*	This study
BCR1662	168, *spoIVB::spec spoIIQ::kan*	This study
BHC77	168, *cotE::kan*	This study
BBK28	168, *spoVID::kan*	[4]
BBK33	168, *safA::kan*	[4]
BJL59	168, *spoIVA::cat*	[4]

Supplementary Table 2. Dimensions of cell wall and coat layers measured from cryo-electron tomograms.

	**Mean thickness** (nm ± SD)[a] (nb of measurements)					
**Strain**	**CW**	**CL**	**LM**	**DM**	**BLL**	**DSL**
ΔspoIVB	26.9 ± 1.3 (36)	12.4 ± 0.7 (18)	31.3 ± 2.4 (24)	17.8 ± 4.9 (24)	8.3 ± 0.8 (24)	7.9 ± 1.0 (24)
ΔcotE[c]	29.9 ± 2.4** (30)	11.2 ± 0.6** (30)	27.4 ± 2.7** (30)	19.7 ± 4.5# (30)	NA	4.8 ± 0.8** (24)

	**Mean distance from the OFM** (nm ± SD)[ab] (nb of measurements)				
**Strain**	**CL**	**LM**	**DM**	**BLL**	**DSL**
ΔspoIVB	NA	NA	31.3 ± 2.4 (24)	55.1 ± 2.7 (24)	81.3 ± 1.4 (24)
ΔcotE[d]	NA	NA	27.4 ± 2.7** (30)	NA	50.2 ± 2.2** (24)

CW, cell wall; CL, crenelated layer; LM, light matrix; DM, dark matrix, BLL, bead-like layer; DSL, dark smooth layer; OFM, outer forespore membrane; NA, not applicable.

[a] Values in nm are presented as mean values ± standard deviations (with numbers of measurements in parentheses).

[b] Measured from the outer face the cytoplasmic membrane to the inner face of the given coat layer.

[c] Two-sided *t* test was performed between corresponding coat layers in *ΔspoIVB* and *ΔcotE* sporangia. #, no statistical difference; *, $P < 0.05$; **, $P < 0.01$. Exact *P* values for significant statistical differences: $P_{(CW)} = 7.03 \times 10^{-5}$; $P_{(CL)} = 7.19 \times 10^{-6}$; $P_{(LM)} = 1.47 \times 10^{-4}$; $P_{(DSL)} = 5.54 \times 10^{-15}$.

[d] Two-sided *t* test was performed between corresponding coat layers in *ΔspoIVB* and *ΔcotE* sporangia. #, no statistical difference; *, $P < 0.05$; **, $P < 0.01$. Exact *P* values for significant statistical differences: $P_{(DM)} = 1.47 \times 10^{-4}$; $P_{(DSL)} = 3.34 \times 10^{-10}$.

Source data are provided as a Source Data file.

Supplementary Table 3. Dimensions of cell wall and coat layers measured from TEM of resin sections.

	**Mean thickness** (nm ± SD)[a] (nb of measurements)						
**Strain**	**CW**	**Thin LM**	**Thin DM**	**Thick LM**	**Thick DM**	**Thin SL**	**Thick SL**
ΔspoIVB	24.5 ± 2.4 (11)	3.2 ± 0.2 (6)	8.9 ± 0.8 (6)	17.8 ± 3.7 (6)	22.5 ± 4.5 (24)	6.0 ± 1.0 (18)	5.2 ± 1.0 (13)
ΔcotE[c]	25.4 ± 1.9 (18)#	3.7 ± 0.5 (12)*	5.1 ± 0.6 (12)**	20.4 ± 3.9 (18)#	20.4 ± 2.2 (18)#	NA	4.7 ± 0.3 (12)#
ΔspoVID[d]	24.2 ± 3.4 (12)#	NA	NA	16.9 ± 2.3 (30)#	20.8 ± 4.0 (30)#	5.3 ± 0.9 (18)*	4.9 ± 0.6 (6)#
ΔsafA[e]	23.1 ± 2.7 (18)#	3.5 ± 0.6 (12)#	5.3 ± 1.3 (12)**	NA	NA	5.0 ± 0.5 (6)#	5.9 ± 1.1 (12)#

	**Mean distance from the OFM** (nm ± SD)[ab] (nb of measurements)					
**Strain**	**Thin LM**	**Thin DM**	**Thick LM**	**Thick DM**	**Thin SL**	**Thick SL**
ΔspoIVB	NA	3.2 ± 0.2 (6)	12.1 ± 0.5 (6)	31.0 ± 3.8 (24)	56.3 ± 6.3 (13)	60.4 ± 2.8 (12)
ΔcotE[f]	NA	3.7 ± 0.5 (12)#	7.8 ± 1.2 (12)#	20.4 ± 3.9 (12)*	NA	56.6 ± 1.5 (12) #
ΔspoVID[g]	NA	NA	NA	17.1 ± 2.3 (24)**	45.8 ± 2.6 (12)**	71.3 ± 3.7 (12)#
ΔsafA[h]	NA	3.5 ± 0.6 (12)#	8.8 ± 0.9 (12)**	NA	NA	NA

CW, cell wall; LM, light matrix; DM, dark matrix, SL, structured layer; OFM, outer forespore membrane; NA, not applicable.

[a] Values in nm are presented as mean values ± standard deviations (with numbers of measurements in parentheses).

[b] Measured from the outer face the OFM to the inner face of the given coat layer. For *ΔspoVID*, we excluded the arch-like structures of the thick dark matrix in our measurements.

[c] Two-sided *t* test was performed between corresponding coat layers in *ΔspoIVB* and *ΔcotE* sporangia. #, no statistical difference; *, $P < 0.05$; **, $P < 0.01$. Exact *P* values for significant statistical differences: $P_{(ThinLM)} = 0.048$; $P_{(ThinDM)} = 4.4 \times 10^{-9}$.

[d] Two-sided *t* test was performed between corresponding coat layers in *ΔspoIVB* and *ΔspoVID* sporangia. #, no statistical difference; *, $P < 0.05$; **, $P < 0.01$. Exact *P* value for significant statistical differences: $P_{(ThinSL)} = 0.049$.
[e] Two-sided *t* test was performed between corresponding coat layers in *ΔspoIVB* and *ΔsafA* sporangia. #, no statistical difference; *, $P < 0.05$; **, $P < 0.01$. Exact *P* value for significant statistical differences: $P_{(ThinDM)} = 1.45 \times 10^{-5}$.
[f] Two-sided *t* test was performed between corresponding coat layers in *ΔspoIVB* and *ΔcotE* sporangia. #, no statistical difference; *, $P < 0.05$; **, $P < 0.01$. Exact *P* value for significant statistical differences: $P_{(ThickDM)} = 0.010$.
[g] Two-sided *t* test was performed between corresponding coat layers in *ΔspoIVB* and *ΔspoVID* sporangia. #, no statistical difference; *, $P < 0.05$; **, $P < 0.01$. Exact *P* values for significant statistical differences: $P_{(ThickDM)} = 5.9 \times 10^{-20}$; $P_{(ThinSL)} = 1.82 \times 10^{-5}$.
[h] Two-sided *t* test was performed between corresponding coat layers in *ΔspoIVB* and *ΔsafA* sporangia. #, no statistical difference; *, $P < 0.05$; **, $P < 0.01$. Exact *P* value for significant statistical differences: $P_{(ThickLM)} = 1.25 \times 10^{-4}$.
Source data are provided as a Source Data file.

Supplementary References

1. Wolff, G. *et al.* Mind the gap: Micro-expansion joints drastically decrease the bending of FIB-milled cryo-lamellae. *J. Struct. Biol.* **208**, 107389 (2019).
2. Moravcová, J., Pinkas, M., Holbová, R. & Nováček, J. Preparation and Cryo-FIB micromachining of Saccharomyces cerevisiae for Cryo-Electron Tomography. *J. Vis. Exp. JoVE* (2021) doi:10.3791/62351.
3. Zeigler, D. R. *et al.* The origins of 168, W23, and other Bacillus subtilis legacy strains. *J. Bacteriol.* **190**, 6983–6995 (2008).
4. Luhur, J. *et al.* A dynamic, ring-forming MucB / RseB-like protein influences spore shape in Bacillus subtilis. *PLoS Genet.* **16**, e1009246 (2020).
