# Supplementary material for: Ultrastructure of macromolecular assemblies contributing to bacterial spore resistance revealed by in situ cryo-electron tomography
Source: Nat Commun. 2024 Feb 14;15:1376. doi: 10.1038/s41467-024-45770-6 (PMC10867305; doi:10.1038/s41467-024-45770-6)
Supplement: Supplementary file 3 — Description of Additional Supplementary Files [file 41467_2024_45770_MOESM3_ESM.pdf]

Description of Additional Supplementary Materials

Supplementary Movie 1. Sequential slices through a representative cryo-electron tomogram of a ΔspoIVB B. subtilis sporulating cell in cross-section view. In this stage-III sporangium, early stages of coat assembly are visible at the surface of the forespore. Related to Figure 1.

Supplementary Movie 2. Sequential slices through a representative cryo-electron tomogram of a ΔcotE B. subtilis sporulating cell in cross-section view. In this stage-III sporangium, the DNA harbors a fibrillary toroid structure in the forespore cytoplasm. Related to Figure 2.

Supplementary Movie 3. 3D rendering model built from the segmentation of the cryo-electron tomogram shown in Supplementary Movie 1. Related to Figure 1.

Supplementary Movie 4. 3D rendering model built from the segmentation of the DNA from the cryo-electron tomogram shown in Supplementary Movie 2. Related to Figure 2.
